# Small G Rac1 is involved in replication cycle of dengue serotype 2 virus in EAhy926 cells via the regulation of actin cytoskeleton

**DOI:** 10.1007/s11427-016-5042-5

**Published:** 2016-04-07

**Authors:** Jing Zhang, Na Wu, Na Gao, Wenli Yan, Ziyang Sheng, Dongying Fan, Jing An

**Affiliations:** 1 Department of Microbiology, School of Basic Medical Sciences, Capital Medical University, Beijing, 100069 China; 2 Center of Epilepsy, Beijing Institute for Brain Disorders, Beijing, 100069 China

**Keywords:** dengue virus, small Rho GTPase Rac1, actin, vascular endothelial cells

## Abstract

Bleeding is a clinical characteristic of severe dengue and may be due to increased vascular permeability. However, the pathogenesis of severe dengue remains unclear. In this study, we showed that the Rac1-microfilament signal pathway was involved in the process of DENV serotype 2 (DENV2) infection in EAhy926 cells. DENV2 infection induced dynamic changes in actin organization, and treatment with Cytochalasin D or Jasplakinolide disrupted microfilament dynamics, reduced DENV2 entry, and inhibited DENV2 assembly and maturation. Rac1 activities decreased during the early phase and gradually increased by the late phase of infection. Expression of the dominant-negative form of Rac1 promoted DENV2 entry but inhibited viral assembly, maturation and release. Our findings demonstrated that Rac1 plays an important role in the DENV2 life cycle by regulating actin reorganization in EAhy926 cells. This finding provides further insight into the pathogenesis of severe dengue.

## INTRODUCTION

Dengue virus (DENV) belongs to the family *Flaviviridae* and is one of the most widespread mosquito-borne human pathogens worldwide, especially in tropical and subtropical countries. Five closely related but antigenically distinct serotypes, DENV1–5, have been identified, including the previously reported serotypes DENV1–4 and the newly discovered serotype DENV-5, which may complicate control efforts (Normile, 2013). An estimated 390 million dengue infections occur each year. Therefore, DENV infection is an important threat to global public health (Bhatt et al., 2013; Fang et al., 2012). However, there is currently no dengue vaccine and anti-viral drug available.

DENV is the causative agent of dengue fever (DF), which manifests as a mild self-limiting febrile illness, i.e., dengue. However, the course of this illness may become complicated and result in the life-threatening clinical form of the disease, i.e., severe dengue. This condition includes dengue hemorrhagic fever (DHF) and dengue shock syndrome (DSS), which are characterized by systemic hemorrhage and increased capillary permeability. However, the mechanism underlying the increased vascular permeability induced by DENV infection is currently unclear. Recently, a morphological study identified hemorrhage, edema and swollen vascular endothelial cells (VECs) in several organs in fatal dengue patients, and viral antigens and negative-strand RNA were detected in VECs, hepatocytes and macrophages (Povoa et al., 2014). This finding indicated that increased vascular permeability and morphological changes of VECs are characteristic pathological features of severe dengue.

The junctional complex in adjacent cells plays important role in maintaining the permeability of VECs. Its role is attributed to a complex association of actin filaments. These filaments are a significant element of endothelial barrier function, which is regulated by linking the filaments to the cytoplasmic tail of junctional adhesive proteins as well as extracellular matrix proteins such as integrins. These complexes then regulate the shape and motility of VECs. In fact, viruses depend on the host cellular machinery to perform their replication life cycle, and a number of cellular components are involved in virus-cell interactions (Angelini et al., 2013; Lyman and Enquist, 2009; Monaghan et al., 2005). Cortical actin filaments directly below the cell membrane form a multifaceted network that provides aphysical barrier to the penetration of viruses and endocytic vesicles (Hanley, 2014; Pfanzagl et al., 2014; Weir et al., 2014). This actin network must be modulated to allow viruses to enter the cell and permit the consequent steps of the viral life cycle, especially intracellular trafficking. Thus, the actin cytoskeleton is one of the first components of the host cell to be disrupted to efficiently establish an infection with a diverse set of viral pathogens (Kallewaard et al., 2005; Sodeik et al., 1997; Stefanovic et al., 2005). Treatment with actin filament inhibitors reportedly affects the entry, replication and release of some viruses via the disruption of actin filament dynamics (Bass et al., 1995; Morrison and McGinnes, 1985). During West Nile virus (WNV) maturation, actin filaments play a key role in viral release (Chu et al., 2003). Furthermore, the actin cytoskeleton plays an indispensable role in influenza virus entry into epithelial cells (Sun and Whittaker, 2007). Our previous study has demonstrated that DENV2 infection induced the rearrangement of microfilaments, and the small GTPase Rac1 played a central role in regulation of the actin cytoskeleton during DENV2 infection in ECV304 cells, a mammalian cell line (Wang et al., 2010). Moreover, we also found that DENV2 infection increased the expression of β3 integrin (Zhang et al., 2007), which may serve as a receptor or co-receptor of DENV2 to mediate viral entry into human microvascular endothelial-1 cells (HMECs-1) and ECV304 cells. The intracellular region of integrins closely associates with actin filaments, and the interplay of β3 integrin and DENV2 might trigger actin reorganization to then affect capillary endothelial integrity. Because ECV304 does not act as an endothelial cell line and the mechanisms of infection of host cells by viruses are cell type-dependent, the necessity of actin and Rac1 for the infection of endothelium-like cells with DENV2 must be confirmed.

Rac1, a member of the Rho family of GTPases is a pivotal component in the regulation of both actin cytoskeletal remodeling and the integrity of intercellular junctions (Bokoch et al., 1996; Edwards et al., 1999; Tolias et al., 1995; Yang et al., 1998). In the absence of vasoactive stimuli, dominant-negative Rac1 affects the function of tight junctions and increases endothelial permeability. Specifically, Rac1 has been suggested to directly regulate these processes. Furthermore, Rac1 negatively regulates clathrin-mediated endocytosis (Lamaze et al., 1996), and some studies have shown that Rac1 is essential for viral infections depending on the regulation of various signal pathways. For example, adenovirus endocytosis requires the assembly of actin via the activation of phosphatidyl inositol kinase-3 (PI3K) and Rac1 (Sanlioglu et al., 2000). Hepatitis B virus (HBV) infection may alter the activity of Rac1, which consequently facilitates HBV replication (Tan et al., 2008). Murine leukemia virus can activate Rac1 in HeLa cells (Krishna and Le Doux, 2006). However, the role of Rac1 activity in the regulation of VEC infection with DENV remains poorly understood.

In the present study, EAhy926 cells, an endothelium-like cell line, was used as a cell model to investigate the effect of the Rac1-microfilament pathway on DENV2 infection. We found that DENV2 infection not only disrupts actin filament dynamics but also induces dynamic changes in the expression of active Rac1. Treatment with actin filament inhibitors elicited diverse effects on various stages of the DENV2 life cycle, including virus entry, assembly, maturation and release. These findings were recapitulated in cells expressing dominant-negative Rac1, suggesting that Rac1 plays an important role in DENV2 infection by regulating microfilaments. Our results not only further elucidate the pathogenesis of severe dengue but also provide further insight into the study of antiviral drugs.

## RESULTS

### Actin filament dynamics are involved in the DENV2 life cycle

Cyto D- or Jas-pretreated EAhy926 cells were infected with DENV2 to investigate the effect of actin dynamics on the DENV2 life cycle. The lysate was collected 1 h after infection for the detection of viral entry. Compared with mock-treated cells, pretreatment with Cyto D and Jas induced approximately 22% and 30% decreases in viral entry (Figure 1A), respectively, and these decreases were significant ($P<0.05$). This finding indicated that actin filament inhibitors inhibited virus entry and that the dynamics were essential for DENV2 entry into EAhy926 cells.

Cyto D- or Jas-pretreated EAhy926 cells were infected with DENV2 to detect the effect of actin dynamics on DENV2 replication. The lysate and supernatant were collected at 4 or 8 h to determine the expression of E protein and the intra- and extracellular viral titers. Except for the decrease in the E protein expression observed in Cyto D-and Jas-pretreated cells 4 h after infection ($P<0.01$ for CytoD-, $P<0.05$ for Jas-), Cyto D or Jas pretreatment did not affect the level of E protein at any other time point (Figure 1B). Moreover, Cyto D and Jas pretreatment decreased the intracellular titers to 66% and 65% at 4 h and to 73% and 66% at 8 h, respectively (Figure 1C). Similarly, Cyto D and Jas pretreatment decreased the extracellular

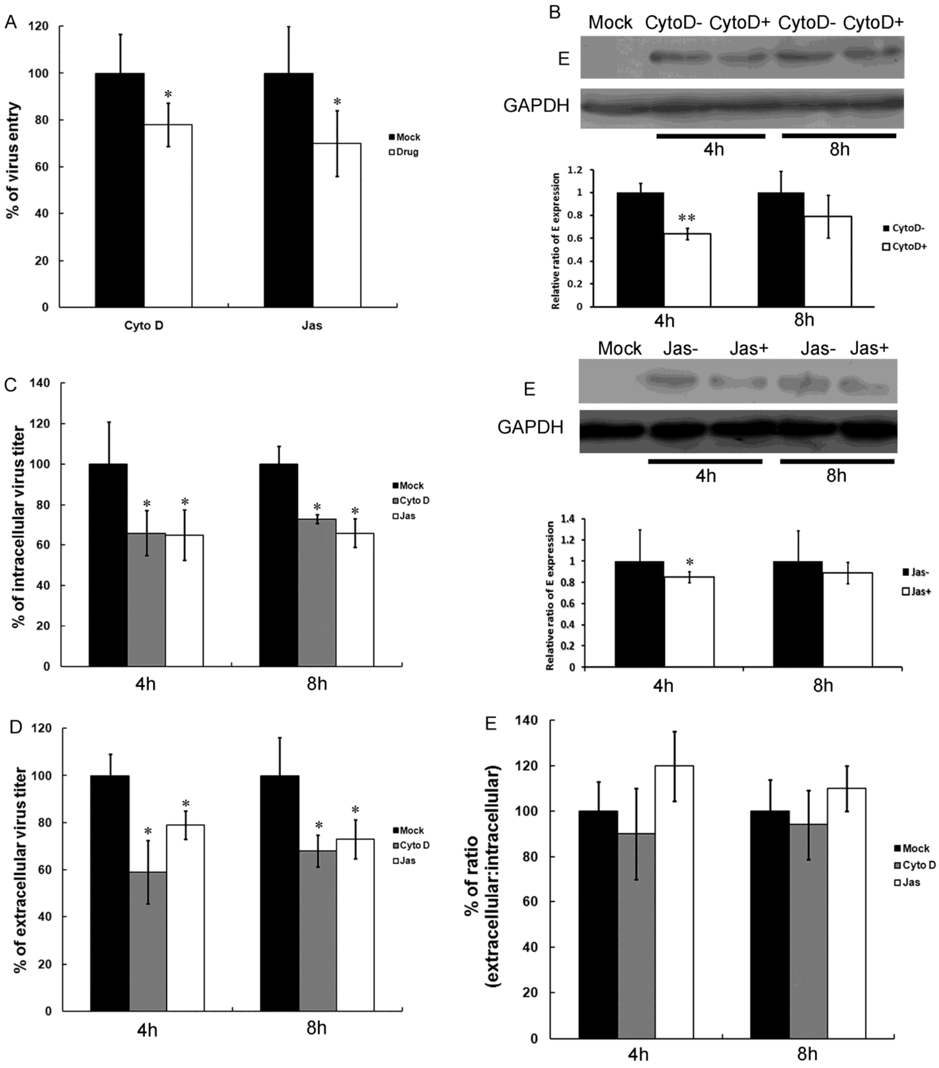

**Figure 1** The role of actin in DENV2 infection. A, The lysates of Cyto D- or Jas- pretreated EAhy926 cells were collected 1 h after DENV2 infection, and the entry of DENV2 was determined with a plaque assay (*n*=3). B, The lysates of Cyto D- or Jas- pretreated EAhy926 cells were collected at 4 and 8 h after DENV2 infection, and the expression levels of E protein were detected with a western blot. The densitometric analysis of E protein levels was expressed as ratios of drugs treated to non-drugs treated. Upper, E protein levels in Cyto D-pretreated cells and their densitometric analysis (*n*=3). Lower, E protein levels in Jas-pretreated cells and their densitometric analysis (*n*=3). GAPDH was used as a loading control. C and D, The lysates and supernatants of Cyto D- or Jas-pretreated EAhy926 cells were collected at 4 and 8 h after infection, and the intra- and extracellular viral titers were determined with a plaque assay. The titers in control cells (DMSO treated) were considered to be 100% (*n*=3). E, The ratios of the extra- to intracellular viral titer are shown as percentages. The data represent the mean ± SD of three independent experiments. *, $P<0.05$ vs. control cells. **, $P<0.01$ vs. control cells.

titers to 59% and 79% at 4 hand to 68% and 73% at 8 h (Figure 1D), respectively. These decreases from the level of mock-treated cells were significant ($P<0.05$) and indicated that the actin dynamics were necessary for DENV2 replication in EAhy926 cells. Moreover, the ratios of extra- to intracellular viral titers after infection in Cyto D- and Jas-pretreated cells were 90% and 120% at 4 h and 94% and 110% at 8 h (Figure 1E), respectively, and did not significantly differ from the titers of mock-treated cells. These results indicated that disrupting the assembly/disassembly actin dynamics likely did not affect viral release from EAhy926 cells.

## Changes in the Rac1-GTP levels after DENV2 infection

The levels of activated Rac1 in DENV2-infected EAhy926 cells were determined with a GST pull-down assay to investigate changes in the Rac1 activity during DENV2 infection. As shown in Figure 2, the levels of GTP-Rac1 (activated Rac1) dynamically changed during DENV2 infection: they markedly decreased at 20 min and 1 h and gradually returned to the control levels at 4 and 8 h and then slightly increased at 12 and 24 h (Figure 2A, $P<0.05$ for 20 min and $P<0.01$ for 1 h). Moreover, activated Rac1 was observed in the cytoplasm 24 h after infection, primarily in the peri-nuclear area (Figure 2B), which indicated that DENV2

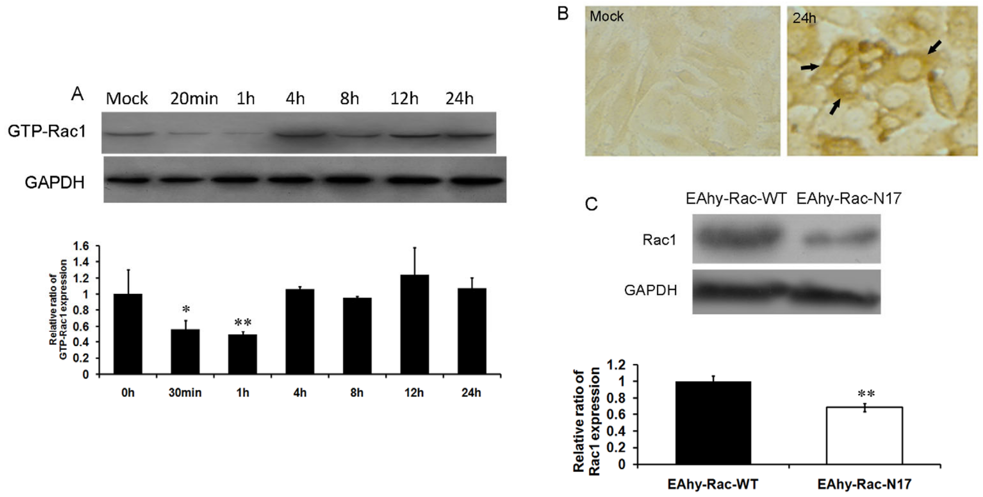

**Figure 2** The changes in Rac1 activity (GTP-Rac1) after DENV2 infection, and identification of the EAhy-Rac-WT and EAhy-Rac-N17 cell lines. A, Upper, the activities of Rac1 (GTP-Rac1) in EAhy926 cells were detected with a western blot at the different indicated time points. GAPDH was used as a loading control. Lower, a densitometric analysis of the western blot results (*n*=3). B, The Rac1 activity (GTP-Rac1) was detected in situ with GST-CRIB (see Material and methods), and activated Rac1 (GTP-Rac1) was observed in the peri-nuclear area of EAhy926 cells (arrow) 24 h after DENV2 infection (×400). C, Upper, the expression level of Rac1 in EAhy-Rac-WT and EAhy-Rac-N17 cells was detected with a western blot. GAPDH was used as a loading control. Lower, a densitometric analysis of the western blot results (*n*=3), expressed as ratio of Rac1 levels in EAhy-Rac-N17 cell to EAhy-Rac-WT. *, $P<0.05$ vs. control cells. **, $P<0.01$ vs. control cells.

infection triggered endogenous Rac1 activation.

### Role of Rac1 in the DENV2 life cycle

We established a cell line in which the expression of Rac1 was down-regulated via the transfection of Rac1 dominant-negative mutant (pRac-N17) to further analyze the effects of Rac1 on DENV2 infection. This cell line was named EAhy-Rac-N17. Simultaneously, we screened a wild-type Rac1 plasmid-transfected cell line, which was named EAhy-Rac-WT. Rac1 activity was significantly reduced in EAhy-Rac-N17 cells (Figure 2C), indicating that our cells could be used in subsequent experiments.

EAhy-Rac-WT and EAhy-Rac-N17 cells were infected with DENV2 to investigate the effect of Rac1 on DENV2 entry. The lysate was collected 1 h after infection to detect the levels of intracellular virus. The DENV2 titer in EAhy-Rac-WT cells was considered to be 100%. As shown in Figure 3A, the percentage of virus entry significantly increased to 233% in EAhy-Rac-N17 cells compared with EAhy-Rac-WT cells, and this increase was significant ($P<0.01$). This result indicated that Rac1 negatively regulates DENV2 entry. Thus, reduced Rac1 activity was required for DENV2 entry into EAhy926 cells.

The lysate and supernatant of DENV2-infected EAhy-Rac-WT and EAhy-Rac-N17 cells were collected at 4 and 8 h after infection to investigate the effect of Rac1 on the assembly and release of DENV2. The levels of E protein and intra- and extracellular viral titers were detected. The expression of E protein in EAhy-Rac-N17 cells did not markedly change 4 and 8 h after infection compared with the levels in EAhy-Rac-WT cells (data not shown). Moreover, the intracellular titers after infection decreased to 74% and 83% in EAhy-Rac-N17 cells at 4 and 8 h compared with the titers in EAhy-Rac-WT cells (Figure 3B, $P<0.05$ for 8 h). The extracellular titers in EAhy-Rac-N17 cells and decreased to 67% and 59% at 4 and 8 h, respectively, compared with the titers in EAhy-Rac-WT cells (Figure 3C, $P<0.01$ for 8 h). The ratios of extra- to intracellular titers in EAhy-Rac-N17 cells were 89% and 68% at 4 h and 8 h after infection, respectively (Figure 3D, $P<0.01$ for 8 h). These results suggested that Rac1 activity was involved in the assembly and release of DENV2.

## DISCUSSION

Asparasitic pathogens, many viruses depend on the components of the host cell to replicate and complete the steps of their life cycle, including viral entry, transportation to the site of replication or the release of progeny virions to the extracellular environment (Angelini et al., 2013; Bass et al., 1995; Garoff et al., 1998; Kallewaard et al., 2005; Lyman and Enquist, 2009; Monaghan et al., 2005; Ott et al., 1996; Ravkov et al., 1998). A variety of viruses have been found to utilize diverse approaches to regulate actin cytoskeletons to create a suitable microenvironment for effectively establishing infection (Chu et al., 2003; Sun and Whittaker, 2007). For example, the vaccinia virus has been shown to induce the formation of actin tails, and its viral particles are propelled on the tips of the actin tails (Frischknecht et al., 1999). In our previous study, we have demonstrated that DENV2 infection induced the reorganization of actin filaments and that are regulator protein, small GTPase Rac1,

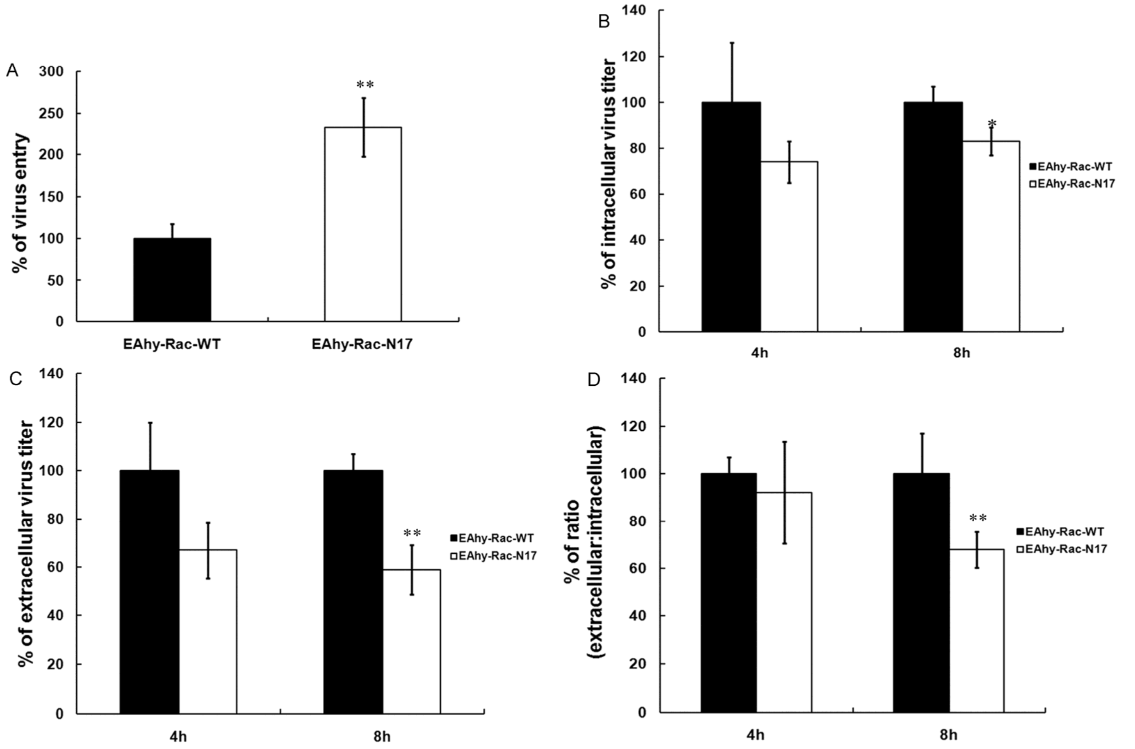

**Figure 3** The effect of Rac1 on DENV2 infection. A, The lysates of EAhy-Rac-WT and EAhy-Rac-N17 cells were collected 1 h after infection, and the entry of DENV2 into the cells was determined with a plaque assay ($n$=3). B and C, The lysates and supernatants of EAhy-Rac-WT and EAhy-Rac-N17 cells were collected 4 and 8 h after infection, and the intra- and extracellular DENV2 titers were determined with a plaque assay. The titers in control cells (EAhy-Rac-WT) were considered to be 100% ($n$=3). D, The ratios of extra- to intracellular viral titer are shown as percentages. The data represent the mean ±SD of three independent experiments. *, $P$<0.05 vs. control cells; **, $P$<0.01 vs. control cells.

was involved in the infection of ECV304 cells with DENV2 (Wang et al., 2010). Here, we provided evidence to further confirm the role of the Rac1-actin pathway in each step of the DENV2 replication cycle in the VEC-like cell line EAhy926.

In this study, DENV2 infection could alter the actin filament dynamics in EAhy926 cells. Moreover, the disruption of actin filaments with Cyto D or Jas inhibited viral entry, assembly and maturation (i.e., decreased E protein levels and intracellular viral titers). The unchanged ratio of the extra- to intracellular viral titer observed may be due to the pre-treatment period (2 h), which is consistent with the findings in our previous study: the repressive effect of actin inhibitors on DENV2 release closely was associated with the treatment time (Wang et al., 2010). Together, these findings indicate that actin is involved in all steps of the DENV2 replication cycle. Cyto D and Jas are known to destabilize actin dynamics via different mechanisms: Cyto D inhibits the polymerization of subunit by binding to the plus-ends of the actin filaments, whereas Jas enhances actin stabilization by inhibiting the depolymerization of actin filaments. Under our experimental condition, the disruption of actin filaments with these two inhibitors elicited similar effects on each step of DENV2 replication cycle, indicating that the dynamic tread milling of actin is important for virus infection.

The actin cytoskeleton is a key factor involved in the endocytosis and phagocytosis of several pathogens, including viruses and bacteria. Previously, we observed that actin was reorganized in DENV2-infected ECV304 cells, and the disruption of actin cytoskeleton with interfering drugs inhibited DENV2 infection (Wang et al., 2010). Chu et al. also identified role for actin in the release of WNV (Chu et al., 2003). In this study, viral entry, assembly and maturation were also repressed when actin was disrupted by treatment with Cyto D or Jas. Because the actin cytoskeleton plays active roles in endocytosis, viral intracellular transportation and release, treatment with Cyto D or Jas likely inhibited DENV2 infection.

Rac1, a member of the Rho GTPase family, is an intrinsically inefficient hydrolyzing enzyme that quickly cycles between its GTP-bound active and GDP-bound inactive forms. Rac1 has been implicated in the negative regulation of clathrin-mediated endocytosis (Lamaze et al., 1996) and also plays a central role in regulating both actin cytoskeletal remodeling and the integrity of intercellular junctions. Recent studies demonstrated that Rac1 plays important roles in many virus infections by regulating actin organization (Jimenez-Baranda et al., 2007; Li et al., 1998; Zhang et al., 2007). For example, adenovirus endocytosis requires the assembly of actin via the activation of Rac1. Integrin β3 regulates DENV2 entry into HMEC-1 cells (Zhang et al., 2007), and Rac1 is required for integrin-dependent cytoskeletal assembly (Li et al., 1998). HIV-1 Env-guided entry is supported by a Filamin A-RhoA-ROCK axis and Arp2/3 complex, both of which are commonly involved in actin cytoskeletal reorganization (Jimenez-Baranda et al., 2007). In this study, DENV2 infection decreased the activities of Rac1 during the early phase of infection, and the role of Rac1 GTPase in the regulation of DENV2 infection was further clearly demonstrated by the increase in virus entry into cells expressing Rac1-N17. These results suggest that viral receptors may initially mediate transient Rac1 inactivation in response to DENV2 binding and/or internalization, and Rac1 might act as a negative regulator of DENV2 entry. Because the Rac1 dominant-negative mutants showed a reduced affinity for nucleotides and cells expressing Rac1-N17 was similar to cells pretreated with Cyto D or Jas, the increase in viral entry observed may be due to the abrogation of downstream active Rac1 signaling, especially the inhibition of actin filament function.

Interestingly Rac1 activity was gradually up-regulated between 4 and 24 h after DENV2 infection, and dominant-negative Rac1 elicited diverse effects on DENV2 replication. Specifically, it increased viral entry during early infection and decreased viral replication during the late stages of infection, which may be due to the synergic effect of various signaling pathways evoked by infection, including host anti-viral responses (Dai et al., 2011; Green et al., 2014; Hishiki et al., 2014; Lei et al., 2013). The aforementioned synergistic effects were further demonstrated by the decreased levels of E protein and intracellular viral titers as well as the decrease in the ratios of extra- and intracellular viral titers observed at the same time point, indicating that Rac1 played an important role in the assembly, maturation and release of DENV2. We previously demonstrated that the transfection of the E gene of DENV2 could trigger the activation of endogenous Rac1. We also showed that the expression of E protein induced the reorganization of the actin cytoskeleton (Wang et al., 2010). These results indicate that viral protein-activated Rac1 is involved in DENV2 infection via the regulation of actin cytoskeleton reorganization. In this study, the Rac1 dominant-negative mutant and Cyto D or Jas treatment elicited similar effects, indicating that activated endogenous Rac1 contributes to the rearrangement of actin filaments during DENV infection and may further influence intercellular junctions and be closely associated with the pathogenesis of severe DF.

In summary, we report here that the Rac1-microfilament pathway plays a complex role in the life cycle of DENV2 replication. During the early phase of infection, decreased Rac1 activity facilitates virus entry, whereas the level of active Rac1 increased during the late phase of infection to inhibit DENV2 assembly, maturation and release. Our results suggest that Rac1 is involved in DENV2 replication via the regulation of actin reorganization. Overall, this study reveals that the Rac1-actin pathway can be co-opted by DENV2 to create a microenvironment that favors the establishment of successful infection. Insight into the DENV2-host interaction not only aids in the design of novel anti-viral drugs but also contributes to our understanding of viral pathogenesis.

## MATERIALS AND METHODS

### Cells and virus

*Aedes albopictus* mosquito (C6/36) cells were grown in RPMI-1640 (GIBCO) containing 10% fetal bovine serum (FBS, GIBCO, USA). A vascular endothelial-like cell line, EAhy926, was grown in Dulbecco's modified Eagle's medium (DMEM, GIBCO) containing 15% FBS. Vero cells were cultured in Eagle's minimum essential medium (MEM, GIBCO) containing 5% FBS.

DENV2 (strain Tr1751), which was isolated from a patient with dengue fever, was propagated in C6/36 cells and stored at −80°C until use. The viral titers were detected with plaque assay using a Vero cell monolayer in 1.2% methylcellulose overlay medium.

### Antibodies and chemicals

Mouse anti-DENV2 polyclonal antibodies (pAbs) were produced in our laboratory. Mouse anti-DENV2 E/NS1 monoclonal antibodies (mAbs) were kindly provided by Dr. Xiaoyan Che (Zhu Jiang Hospital, Southern Medical University, Guangzhou, China). Rabbit anti-GAPDH pAbs were produced by Bioworld Technology (Nanjing). Horseradish peroxidase (HRP)-conjugated goat anti-mouse immunoglobulin (IgG) and HRP-conjugated goat anti-rabbit IgG were purchased from Ding Guo Chang Sheng Biotechnology (Beijing). FITC-conjugated goat anti-mouse IgG, Cy3-conjugated goat anti-mouse IgG, Cytochalasin D (Cyto D) and Jasplakinolide (Jas) were purchased from Sigma (USA). Anti-Rac1 mAb was purchased from Cytoskeleton (USA), and anti-GST pAbs were purchased from TransGen Biotech (Beijing). Lipofectamine was purchased from Invitrogen (USA).

### Effect of microfilament inhibitors on DENV2 infection

Cyto D and Jas, dissolved in dimethyl sulfoxide (DMSO), were stored at −20°C and used at concentrations of 2 μmol $L^{-1}$ and 50 nmol $L^{-1}$, respectively, as determined with 3-(4,5-dimethylthiazol-2-yl)-diphenyltetrazolium bromide (MTT, Sigma). EAhy926 cells were grown in 6-well culture plates and pretreated with DMEM containing 2 μmol $L^{-1}$ of Cyto D, 50 nmol $L^{-1}$ of Jas, or 0.1% of DMSO (mock- treatment, as control) at 37°C for 2 h. The cells were then infected with DENV2 (MOI=10) at 37°C for 1 h. Subsequently, the supernatant and/or cell lysate were collected at different indicated time points to determine the effect of the above inhibitors on the DENV2 life cycle.

To investigate effect of Cyto D or Jas on DENV2 entry, the inoculated viruses were removed 1h after infection, and the cells were treated with acid glycine (pH 3.0) for 2 min to inactivate extracellular virus. The cell lysates were then collected, and the viral titers were determined with plaque assays using Vero cells. The titer of the mock treatment (DMSO control) was considered to be 100%.

To investigate the effects of CytoD or Jas on DENV2 assembly and release, DENV2-infected cells were cultured in medium containing 2 μmol $L^{-1}$ of CytoD or 50 nmol $L^{-1}$ of Jas, and the supernatant and lysate were collected 4 and 8 h after infection. The E protein was detected by western blot, whereas extracellular and intracellular virus levels were detected with a plaque assay. Three independent experiments were performed for each group (CytoD, Jas and control) and each time point. The titer of the mock treatment (DMSO control) was considered to be 100%.

## Purification of GST-CRIB

The pGEX-6p-CRIB plasmid was constructed as described previously (Wang et al., 2010). Briefly, the cDNA sequence of the CRIB domain was amplified from mouse brains and cloned into the pGEX-6p-1. Subsequently, *Escherichia coli* transformed with pGEX-6p-CRIB was grown in LB medium containing 0.1 mg $mL^{-1}$ ampicillin at 37°C for 24 h. The culture was then diluted 1∶50 with fresh LB medium containing 0.1 mg $ml^{-1}$ ampicillin and incubated at 37°C for 3 h. Thereafter, the cells were incubated with 1 mmol $L^{-1}$ IPTG (isopropyl-beta-D-thiogalactopyranoside) at 28°C for 24 h, followed by centrifugation at 2,668×*g* for 10 min and re-suspension in phosphate buffered saline (PBS). After lysing the cells by sonication and centrifugation at 15,365×*g* for 20 min, the soluble fraction was incubated with glutathione Sepharose 4B beads at 4°C for 24 h. The beads were washed three times with PBS. GST-CRIB was then harvested and kept at 4°C in the presence of sodium azide and a cocktail of protease inhibitors.

## *In situ* detection of Rac1 activity (Rac1-GTP) after DENV2 infection

EAhy926 cells grown on coverslips were infected with DENV2 (MOI=10). 24 h after infection, the cells were fixed with 4% paraformaldehyde (in PBS) and then treated with 0.2% Triton X-100 (in PBS) followed by incubation with 1% bovine serum albumin (BSA in PBS) at 37°C for 30 min. The cells were then incubated with GST-CRIB (10 μg $mL^{-1}$) at 4°C overnight. Subsequently, the level of GST-CRIB was detected with an anti-GST antibody (1∶100) and HRP-conjugated secondary antibody.

## Pull-down assay for Rac1-GTP

GTP-Rac1 was identified by CRIB. Briefly, EAhy926 cells (approximately $5\times10^6$) were seeded and starved of serum for approximately 24 h before being infected with DENV2 (MOI=10). At various time points after infection, the cells were washed twice with ice-cold PBS and lysed in 500 μL of lysis buffer (RIPA lysis buffer). The lysate was incubated with glutathione beads containing GST-CRIB for 90 min at 4°C, and the level of Rac1-GTP was then determined by western blot.

## Generation of EAhy926 cells stably expressing Rac1-WT and Rac1-N17

The pRe-WT and pRe-N17 plasmids, which express wild-type Rac1 and a dominant-negative mutant of Rac1, respectively, were constructed as described previously (Wang et al., 2010). EAhy926 cells were transfected with 4 μg of pRe-WT or pRe-N17 using Lipofectamine 2000 (Life Technologies, USA) according to the manufacturer's instructions. 48 h after transfection, the medium was replaced with fresh DMEM containing 15% FBS and 0.8 mg $mL^{-1}$ G418, and the cells were continuously cultured for 2–3 d until the mock-treated cells died. The cells were then incubated with fresh DMEM containing 15% FBS and 0.4 mg $mL^{-1}$ G418 for several days to yield cells carrying pRe-WT or pRe-N17; these cells were named EAhy-Rac-WT and EAhy-Rac-N17, respectively.

## Effect of Rac1 on DENV2 infection

EAhy-Rac-WT and EAhy-Rac-N17 were infected with DENV2 (MOI=10) at 37°C for 1 h. The supernatant and cell lysate were then collected at different time points after infection to measure the protein levels and viral titers with western blot and plaque assays, respectively. The viral titers in EAhy-Rac-WT cells were considered to be 100%. Three independent experiments were performed for each cell line and each time point.

## Statistical analysis

The statistical analyses were performed with SPSS 16.0. The quantitative data were compared between two groups using a *t*-test. Differences between groups were considered to be significant at $P<0.05$.

**Compliance and ethics** *The author(s) declare that they have no conflict of interest.*

***Acknowledgements*** *The authors wish to thank Professor Xiaoyan Che (Zhu Jiang Hospital, Southern Medical University, Guangzhou, China )for her kind gift of the mAb against E protein. This work was supported by the National Key Programs on Basic Research of China (2011CB504703), the National Natural Science Foundations of China (81301435, 81471957, 81271839, 81401676) and Beijing Natural Science Foundation (7144194).*

Angelini, M.M., Akhlaghpour, M., Neuman, B.W., and Buchmeier, M.J. (2013). Severe acute respiratory syndrome coronavirus nonstructural proteins 3, 4, and 6 induce double-membrane vesicles. MBio doi:

10.1128/mBio.00524-13.
Bass, D.M., Baylor, M., Chen, C., and Upadhyayula, U. (1995). Dansylcadaverine and cytochalasin D enhance rotavirus infection of murine L cells. Virology 212, 429–437.
Bhatt, S., Gething, P.W., Brady, O.J., Messina, J.P., Farlow, A.W., Moyes, C.L., Drake, J.M., Brownstein, J.S., Hoen, A.G., Sankoh, O., Myers, M.F., George, D.B., Jaenisch, T., Wint, G.R., Simmons, C.P., Scott, T.W., Farrar, J.J., and Hay, S.I. (2013). The global distribution and burden of dengue. Nature 496, 504–507.
Bokoch, G.M., Vlahos, C.J., Wang, Y., Knaus, U.G., and Traynor-Kaplan, A.E. (1996). Rac GTPase interacts specifically with phosphatidylinositol 3-kinase. Biochem J 315 ( Pt 3), 775–779.
Chu, J.J., Choo, B.G., Lee, J.W., and Ng, M.L. (2003). Actin filaments participate in West Nile (Sarafend) virus maturation process. J Med Virol 71, 463–472.
Dai, J., Pan, W., and Wang, P. (2011). ISG15 facilitates cellular antiviral response to dengue and west nile virus infection *in vitro*. Virol J 8, 468.
Edwards, D.C., Sanders, L.C., Bokoch, G.M., and Gill, G.N. (1999). Activation of LIM-kinase by Pak1 couples Rac/Cdc42 GTPase signalling to actin cytoskeletal dynamics. Nat Cell Biol 1, 253–259.
Fang, X., Hu, Z., Shang, W., Zhu, J., Xu, C., and Rao, X. (2012). Genetic polymorphisms of molecules involved in host immune response to dengue virus infection. FEMS Immunol Med Microbiol 66, 134–146.
Frischknecht, F., Moreau, V., Rottger, S., Gonfloni, S., Reckmann, I., Superti-Furga, G., and Way, M. (1999). Actin-based motility of vaccinia virus mimics receptor tyrosine kinase signalling. Nature 401, 926–929.
Garoff, H., Hewson, R., and Opstelten, D.J. (1998). Virus maturation by budding. Microbiol Mol Biol Rev 62, 1171–1190.
Green, A.M., Beatty, P.R., Hadjilaou, A., and Harris, E. (2014). Innate immunity to dengue virus infection and subversion of antiviral responses. J Mol Biol 426, 1148–1160.
Hanley, J.G. (2014). Actin-dependent mechanisms in AMPA receptor trafficking. Front Cell Neurosci 8, 381.
Hishiki, T., Han, Q., Arimoto, K., Shimotohno, K., Igarashi, T., Vasudevan, S.G., Suzuki, Y., and Yamamoto, N. (2014). Interferon-mediated ISG15 conjugation restricts dengue virus 2 replication. Biochem Biophys Res Commun 448, 95–100.
Jimenez-Baranda, S., Gomez-Mouton, C., Rojas, A., Martinez-Prats, L., Mira, E., Ana Lacalle, R., Valencia, A., Dimitrov, D.S., Viola, A., Delgado, R., Martinez, A.C., and Manes, S. (2007). Filamin-A regulates actin-dependent clustering of HIV receptors. Nat Cell Biol 9, 838–846.
Kallewaard, N.L., Bowen, A.L., and Crowe, J.E., Jr. (2005). Cooperativity of actin and microtubule elements during replication of respiratory syncytial virus. Virology 331, 73–81.
Krishna, D., and Le Doux, J.M. (2006). Murine leukemia virus particles activate Rac1 in HeLa cells. Biochem Biophys Res Commun 345, 1184–1193.
Lamaze, C., Chuang, T.H., Terlecky, L.J., Bokoch, G.M., and Schmid, S.L. (1996). Regulation of receptor-mediated endocytosis by Rho and Rac. Nature 382, 177–179.
Lei, S., Tian, Y.P., Xiao, W.D., Li, S., Rao, X.C., Zhang, J.L., Yang, J., Hu, X.M., and Chen, W. (2013). ROCK is involved in vimentin phosphorylation and rearrangement induced by dengue virus. Cell Biochem Biophys 67, 1333–1342.
Li, E., Stupack, D., Bokoch, G.M., and Nemerow, G.R. (1998). Adenovirus endocytosis requires actin cytoskeleton reorganization mediated by Rho family GTPases. J Virol 72, 8806–8812.
Lyman, M.G., and Enquist, L.W. (2009). Herpesvirus interactions with the host cytoskeleton. J Virol 83, 2058–2066.
Monaghan, P., Gold, S., Simpson, J., Zhang, Z., Weinreb, P.H., Violette, S.M., Alexandersen, S., and Jackson, T. (2005). The alpha(v)beta6 integrin receptor for Foot-and-mouth disease virus is expressed constitutively on the epithelial cells targeted in cattle. J Gen Virol 86, 2769–2780.
Morrison, T.G., and McGinnes, L.J. (1985). Cytochalasin D accelerates the release of Newcastle disease virus from infected cells. Virus Res 4, 93–106.
Normile, D. (2013). Tropical medicine. Surprising new dengue virus throws a spanner in disease control efforts. Science 342, 415.
Ott, D.E., Coren, L.V., Kane, B.P., Busch, L.K., Johnson, D.G., Sowder, R.C., 2nd, Chertova, E.N., Arthur, L.O., and Henderson, L.E. (1996). Cytoskeletal proteins inside human immunodeficiency virus type 1 virions. J Virol 70, 7734–7743.
Pfanzagl, B., Andergassen, D., Edlmayr, J., Niespodziana, K., Valenta, R., and Blaas, D. (2014). Entry of human rhinovirus 89 via ICAM-1 into HeLa epithelial cells is inhibited by actin skeleton disruption and by bafilomycin. Arch Virol 159, 125–140.
Povoa, T.F., Alves, A.M., Oliveira, C.A., Nuovo, G.J., Chagas, V.L., and Paes, M.V. (2014). The pathology of severe dengue in multiple organs of human fatal cases: histopathology, ultrastructure and virus replication. PLoS One 9, e83386.
Ravkov, E.V., Nichol, S.T., Peters, C.J., and Compans, R.W. (1998). Role of actin microfilaments in Black Creek Canal virus morphogenesis. J Virol 72, 2865–2870.
Sanlioglu, S., Benson, P.K., Yang, J., Atkinson, E.M., Reynolds, T., and Engelhardt, J.F. (2000). Endocytosis and nuclear trafficking of adeno-associated virus type 2 are controlled by rac1 and phosphatidylinositol-3 kinase activation. J Virol 74, 9184–9196.
Sodeik, B., Ebersold, M.W., and Helenius, A. (1997). Microtubule-mediated transport of incoming herpes simplex virus 1 capsids to the nucleus. J Cell Biol 136, 1007–1021.
Stefanovic, S., Windsor, M., Nagata, K.I., Inagaki, M., and Wileman, T. (2005). Vimentin rearrangement during African swine fever virus infection involves retrograde transport along microtubules and phosphorylation of vimentin by calcium calmodulin kinase II. J Virol 79, 11766–11775.
Sun, X., and Whittaker, G.R. (2007). Role of the actin cytoskeleton during influenza virus internalization into polarized epithelial cells. Cell Microbiol 9, 1672–1682.
Tan, T.L., Fang, N., Neo, T.L., Singh, P., Zhang, J., Zhou, R., Koh, C.G., Chan, V., Lim, S.G., and Chen, W.N. (2008). Rac1 GTPase is activated by hepatitis B virus replication—involvement of HBX. Biochim Biophys Acta 1783, 360–374.
Tolias, K.F., Cantley, L.C., and Carpenter, C.L. (1995). Rho family GTPases bind to phosphoinositide kinases. J Biol Chem 270, 17656–17659.
Wang, J.L., Zhang, J.L., Chen, W., Xu, X.F., Gao, N., Fan, D.Y., and An, J. (2010). Roles of small GTPase Rac1 in the regulation of actin cytoskeleton during dengue virus infection. PLoS Negl Trop Dis doi: 10.1371/journal.pntd.0000809.
Weir, D.L., Laing, E.D., Smith, I.L., Wang, L.F., and Broder, C.C. (2014). Host cell virus entry mediated by Australian bat lyssavirus G envelope glycoprotein occurs through a clathrin-mediated endocytic pathway that requires actin and Rab5. Virol J 11, 40.
Yang, N., Higuchi, O., Ohashi, K., Nagata, K., Wada, A., Kangawa, K., Nishida, E., and Mizuno, K. (1998). Cofilin phosphorylation by LIM-kinase 1 and its role in Rac-mediated actin reorganization. Nature 393, 809–812.
Zhang, J.L., Wang, J.L., Gao, N., Chen, Z.T., Tian, Y.P., and An, J. (2007). Up-regulated expression of beta3 integrin induced by dengue virus serotype 2 infection associated with virus entry into human dermal microvascular endothelial cells. Biochem Biophys Res Commun 356, 763–768.

**Open Access** This article is distributed under the terms of the Creative Commons Attribution License which permits any use, distribution, and reproduction in any medium, provided the original author(s) and source are credited.
